# The Quantum Binding Problem in the Context of Associative Memory

**DOI:** 10.1371/journal.pone.0162312

**Published:** 2016-09-07

**Authors:** Andreas Wichert

**Affiliations:** Department of Computer Science and Engineering, INESC-ID & Instituto Superior Técnico, Universidade de Lisboa, Porto Salvo, Portugal; University of the West of England, UNITED KINGDOM

## Abstract

We present a method to solve the binding problem by using a quantum algorithm for the retrieval of associations from associative memory during visual scene analysis. The problem is solved by mapping the information representing different objects into superposition by using entanglement and Grover’s amplification algorithm.

## Introduction

Quantum machine learning by a quantum computer can take logarithmic time in the number of vectors and their dimension [1–4]. This time is a significant exponential speed-up over classical algorithms; however, such a speed-up requires quantum input and quantum output [3]. In the absence of quantum input, the data must be read, which results in a linear time complexity. We can use Grover’s algorithm to achieve a quadratic speed-up without requiring the output states to be quantum states. However, most quantum machine learning algorithms and quantum associative memories that are based on Grover’s algorithm suffer from the unsolved reading destruction problem (RD problem) [3–5]:

- The reading problem: The amplitude distribution of a quantum state is initialized by reading *N* data points. Although the existing quantum algorithm requires only $O(\sqrt{N})$ steps and is faster than the classical algorithms, *N* data points must be read. Hence, the complexity of the algorithm does not improve and is $O\left(N\right)=O\left(N\right)+O\left(\sqrt{N}\right)$.
- The destruction problem: A quantum associative memory [6–8] for *N* data points of dimension *m* requires only *m* or fewer units (quantum bits). However, this memory can be queried only once because of the collapse during measurement (destruction); hence, quantum associative memory does not have any advantages over classical memory.

We identify a quantum algorithm with a Wilshaw’s associative memory model [9, 10] that does not suffer from the RD problem. The visual (sub-symbolic) variable binding algorithm for Wilshaw’s associative memory involves intensive computation because it corresponds to a combinatorial problem [11, 12]. We suggest some mechanisms based on quantum computation to reduce this high complexity and present a hybrid architecture from the quantum computation perspective. In the proposed architecture, different objects are mapped into superposition representing different combinations. Wilshaw’s associative memory performs the familiarity discrimination task [13] that can be efficiently represented by a quantum oracle. Grover’s algorithm considerably improves the time complexity. The main contributions of this interdisciplinary paper are the relation of sub-symbolic variable binding, the usage of Wilshaw’s associative memory for familiarity discrimination and its integration into a hybrid quantum computation architecture.

This paper comprises a classical section that reviews previously published work [11, 12, 14] related to the sub-symbolic variable binding approach and an integration section that describes the new quantum computational approach.

## Materials and Methods

### Sub-symbolic Binding

The binding problem determines a method to connect all of the physically separated fragments of a complex object to enable them to be processed as a whole by an agent. For example, a red block is obviously a different object from a blue block. The binding problem can be divided into two subproblems: the segregation problem and the combination problem. The segregation problem involves the determination of a method to segregate the elements in an input such that they represent objects that can also be features. The combination problem involves the determination of the elements that represent an object or a category. In sub-symbolic binding, the elements are represented by sub-symbols. The sub-symbolic representation often corresponds to a pattern that mirrors the manner in which the biological sense organs describe the world. Patterns are represented by vectors. Thus, the vectors correspond to sub-symbols. An example of a sub-symbolic binding problem is the definition of a category of objects in a visual scene [11, 12].

#### Sub-symbolic combination problem

Suppose 7 objects were recognized in the visual scene. We represent the 7 objects at various positions in the scene by the symbols *A*, *B*, *C*, *D*, *E*, *F*, *G*. The task is the identification of a category that is formed by the objects represented by the set *B*, *C*, *G* [11, 12, 14]. We determine whether each of the symbols *B*, *C*, *G* is present in the set that represents the scene. We also verify whether a set representing a category is a subset of the set representing a scene. This task is described by proto logic sets [14]. Proto logic operates on sets; it verifies whether a subset is present in a certain set. The proto logic task seems trivial in the case of sets and symbols. However, if the category (set of sub-symbols) is stored in an associative memory, the task is non-trivial and is an example of the combination problem. The combination problem determines a method to combine individual objects or features into a single category. In an associative memory, we do not have direct access to the stored information.

An associative memory operates on vectors of fixed dimensions. Two of these vectors are always associated; this process of association is called learning. The first vector is called the address vector, and the second vector is called the retrieved vector. After the learning process, the address vector is presented to the associative memory, and the retrieved vector is determined. This process is called association. A distinction exists between heteroassociation and auto-association. An auto-association is present if the retrieved vector represents the reconstruction of the faulty address vector. A heteroassociation is present if the retrieved vector is different from the address vector. In our model, we store auto-associations, i.e., the address vector is the same as the retrieved vector. After a retrieved vector is determined, the similarity between the determined retrieved vector and the address vector is calculated. A greater similarity indicates a higher probability of the corresponding address vector being stored in the associative memory.

A set of objects (a category) is represented by a vector created by concatenating the sub-vectors that represent the objects. For *M* sub-vectors, the number of possible orderings of the corresponding sub-vectors is *M*!. To verify whether a set of *M* sub-vectors representing a category is a subset of the set of *N* sub-vectors representing a scene, there are

$$\begin{array}{c} L=Perm\left(N,M\right)=\frac{N!}{(N-M)!} \end{array}$$ (1)

**Fig 1:**
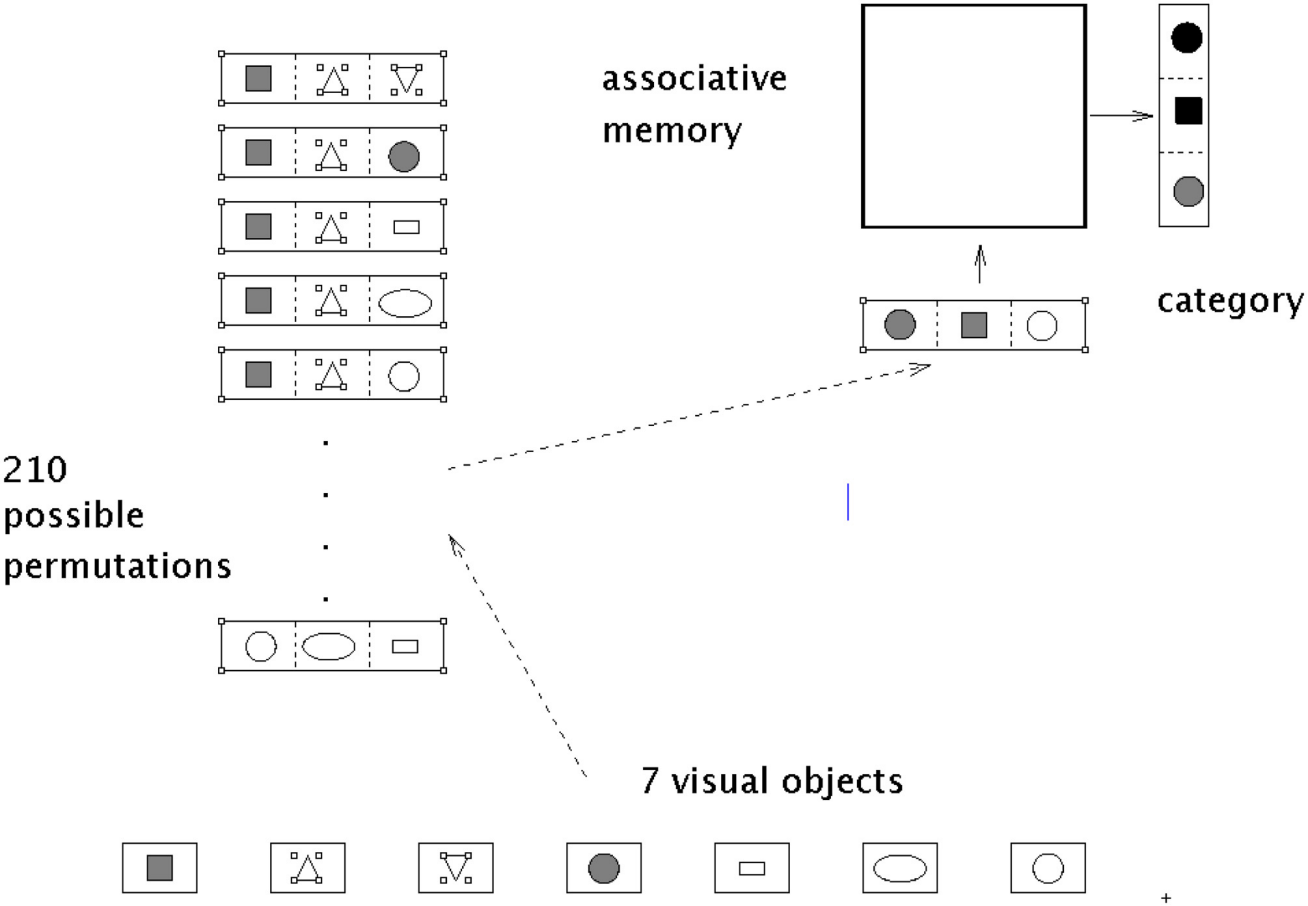
Retrieval phase. In the retrieval phase *L* permutations are formed. Each permutation represents a address vector **x**_*i*_, *i* ∈ {1, …, *L*}. Some of the address vectors represent a category, given that the determined retrieved vector is similar to the address vector.

*L* possible orderings [11, 12, 15]. For *N* = 7 and *M* = 3, we must pose *L* = 210 queries to the associative memory, as shown in Fig 1.

The combination problem leads to an combinatorial explosion for large *N* and *M* values, for example for *N* = 100 and *M* = 4 the value 9.41094 ⋅ 10^7^. From the *L* permutations only the familiar patterns represent visual categories that were learned.

### Wilshaw’s Associative Memory

**Fig 2:**
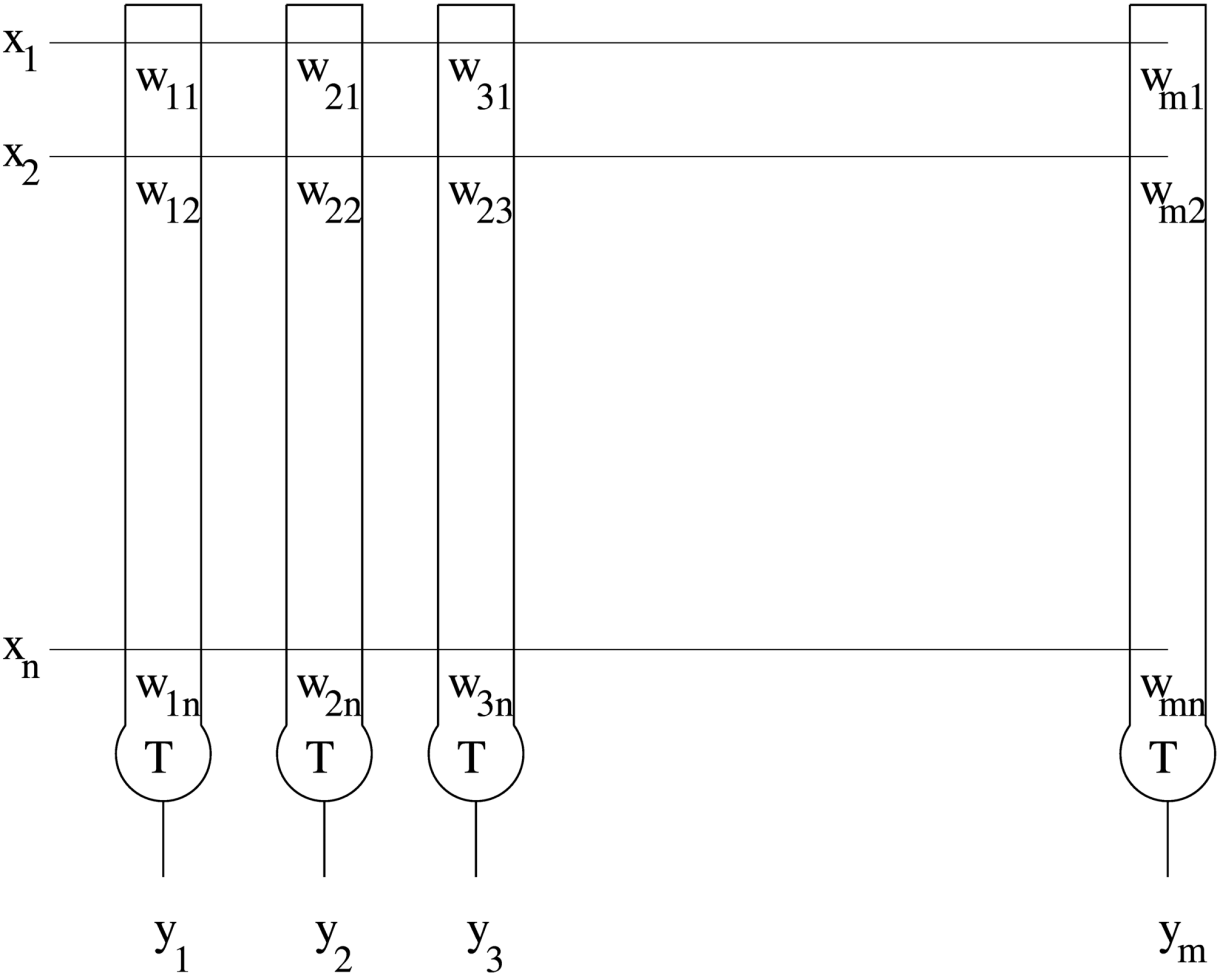
The associative memory. The associative memory is composed of a cluster of units.

An example for an associative memory is the formal neural net model that integrates the assembly concept [9, 10], also called Lernmatrix or Wilshaw’s associative memory. The biological and mathematical aspects of the Wilshaw’s associative memory were studied by Wilshaw and Palm [9, 10, 16]. It was shown that Donald Hebb’s hypothesis of cell assemblies as a biological model of internal representation of events and situations in the cerebral cortex corresponds to the formal Wilshaw’s associative memory model. The Lernmatrix [17, 18] is composed of a cluster of units which represent a simple model of a real biological neuron. The unit is composed of weights which correspond to the synapses and dendrites in the real neuron. They are described by *w*_*ij*_ in Fig 2. *T* is the threshold of the unit. We call the Lernmatrix simply “associative memory” if no confusion with other models is possible. Two pairs of binary vectors are associated, this process of association is called learning. The first of the two vectors is called the address vector and the second, the retrieved vector. After learning, the address vector is presented to the Lernmatrix and the retrieved vector is determined.

#### Learning

In the initialization phase of the associative memory, no information is stored. Because the information is represented in weights, they are all initially set to zero. In the learning phase, pairs of binary vector are associated. Let **x** be the address vector and **y** the retrieved vector, the learning rule is:

$$\begin{array}{c} w_{ij}^{new}=\left\{\begin{array}{cc} 1 & if\;\;y_{i}\cdot x_{j}=1\\ w_{ij}^{old} & \text{otherwise} \end{array}\right. \end{array}$$ (2)

#### Retrieval

In the *one-step* retrieval phase of the associative memory, a fault tolerant answering mechanism recalls the appropriate answer vector for a address vector **x**. For the presented address vector **x**, the most similar learned **x**^*l*^ address vector regarding the Hamming distance is determined and the appropriate retrieved vector **y** is identified. For the retrieval rule, the knowledge about the correlation of the components is sufficient. The retrieval rule for the determination of the retrieved vector **y** is:

$$\begin{array}{c} y_{i}=\left\{\begin{array}{cc} 1 & \;\sum_{j=1}^{n}w_{ij}x_{j}\geq T\;\\ 0 & \;\text{otherwise.} \end{array}\right. \end{array}$$ (3)

where *T* is the threshold of the unit. The threshold is set as proposed by [19] to the maximum of the sums $\overset{n}{\underset{j=1}{\sum }}w_{ij}x_{j}$:

$$\begin{array}{c} T:=\max_{1\leq i\leq B}\left\{\sum_{j=1}^{n}w_{ij}x_{j}\right\}. \end{array}$$ (4)

Only the units which are maximal correlated with the address vector are set to one.

### Familiarity Discrimination

For the computation of the reliability of the the answer for the heteroassociation a backward projection of the associative memory is required [12]. The backward projection corresponds to a bidirectional associative memory (BAM) [20]. This time the learned matrix is cued with the retrieved vector and the best address vector is retrieved. Formally, **y** is the address vector, and the retrieved vector which should be determined is **x**^*l*^. The categorization rule for the determination of the retrieved vector **x**^*l*^ is:

$$\begin{array}{c} x_{j}^{l}=\left\{\begin{array}{cc} 1 & \;\sum_{i=1}^{m}w_{ij}y_{i}\geq T^{*}\;\\ 0 & \;\text{otherwise.} \end{array}\right. \end{array}$$ (5)

This means that the synaptic matrix used is a transposition of the matrix which is used for the forward projection. T* is the threshold of the unit. The threshold is set to the maximum sum $\overset{m}{\underset{j=1}{\sum }}w_{ij}y_{j}$:

$$\begin{array}{c} T^{*}:=\max_{1\leq j\leq A}\left\{\sum_{i=1}^{m}w_{ji}y_{i}\right\}. \end{array}$$ (6)

Let **x** be the question vector and **y** the retrieved vector that was determined by the associative memory for example by a part of the associative memory. First, the vector **x**^*l*^ which belongs to the vector **y** is determined. These two vectors form together a vector pair **x**^*l*^ **y** which is stored in the associative memory. It was either created by learning, **x**^*l*^ and **y** were learned together, or created through overlap with other already learned vector pairs. The vector **x**^*l*^ is determined by a backward projection of the vector **y**. In the second step, the similarity of the stored address vector **x**^*l*^ to the actually presented vector **x** is determined. The greater the similarity of the vector **x**^*l*^ to the vector **x**, the more reliable the retrieved vector **y**. We can measure the similarity by the Hamming distance function

$$\begin{array}{c} d_{1}\left(x,x^{l}\right)=∥x-x^{l}{∥}_{1}=|x_{1}-x_{1}^{l}\left|+\right|x_{2}-x_{2}^{l}\left|+\cdots +\right|x_{m}-x_{m}^{l}| \end{array}$$ (7)

or by the scalar product

$$\begin{array}{c} \left\langle x|x^{l}\right\rangle =\cos\omega \cdot ∥x∥\cdot ∥x^{l}∥, \end{array}$$ (8)

that measure of the projection of one vector onto another.

For auto-association the task there is no need for a backward projection. In the case of auto-association *n* = *m*. We can measure the similarity by the scalar product with

$$\begin{array}{c} y=W\cdot x \end{array}$$ (9)

and

$$\begin{array}{c} \left\langle x|y\right\rangle \end{array}$$ (10)

since *W* is symmetric with *n* = *m*. This is equivalent to the quadratic form

$$\begin{array}{c} net=x^{⊤}\cdot W\cdot x=\sum_{i=1}^{n}\sum_{j=1}^{n}w_{ij}\cdot x_{i}\cdot x_{j}. \end{array}$$ (11)

The quadratic form can be as well be interpreted as the energy function [13]

$$\begin{array}{c} H=-\sum_{i=1}^{n}\sum_{j=1}^{n}w_{ij}\cdot x_{i}\cdot x_{j}. \end{array}$$ (12)

The threshold operation to determine similarity *sim* is applied to the scalar value *net*,

$$\begin{array}{c} sim=\left\{\begin{array}{cc} 1 & \;net\geq t\;\\ 0 & \;\text{otherwise.} \end{array}\right. \end{array}$$ (13)

with the threshold

$$\begin{array}{c} t:=T^{2} \end{array}$$ (14)

*sim* = 1 indicates that the question vector **x** was stored in the associative memory, *sim* = 0 that is was not. This operation called the familiarity discrimination, in familiarity discrimination there is no need per se to extract the whole answer pattern [13]. In the following we will only preform auto-association.

### Visual Scene Coding

The visual system recognizes objects in an image. It was suggested [21] that the brain includes two mechanisms for visual categorization [22]: one for the representation of the object and the other for the representation of the localization [23]. The first mechanism is called the *what* pathway and is located in the temporal lobe. The second mechanism is called the *where* pathway and is located in the parietal lobe. According to this division, the identity of a visual object can be coded apart from its location. A visual scene can be either represented by an image or by objects and their position in the visual field. Objects are represented by pictograms together with their corresponding position in the image. This is a simple form of structured and compressed representation of a mental image.

**Fig 3:**
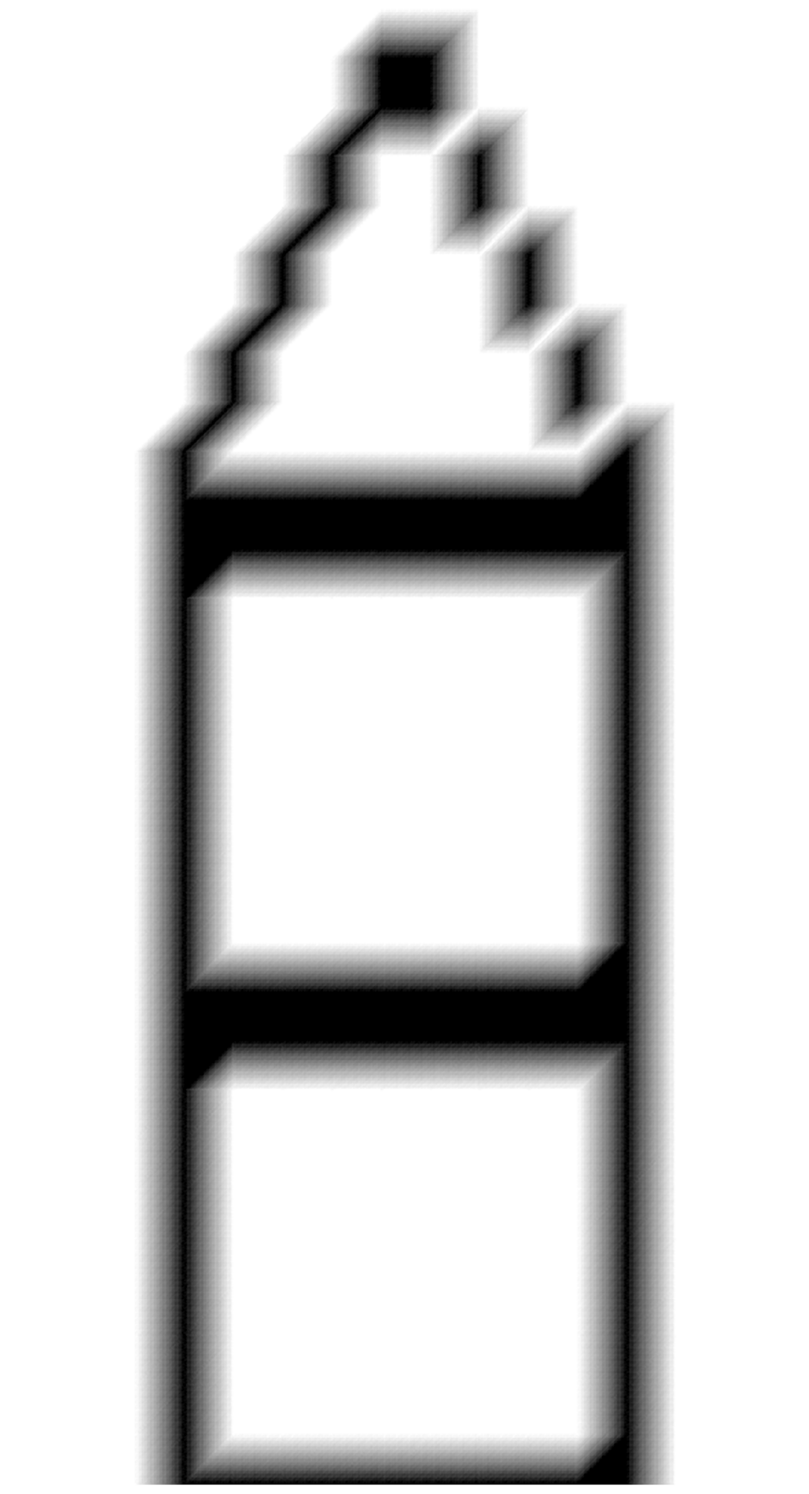
Category. Category “tower” in the blockworld. Blocks can be placed in different positions and picked up and set down. There are two different classes of blocks: cubes and pyramids.

The definition of a visual category (see Fig 3) is motivated by the verbal category definition that is a set of prototypical features [24], such as red, round and sweet [25, 26].

**Fig 4:**
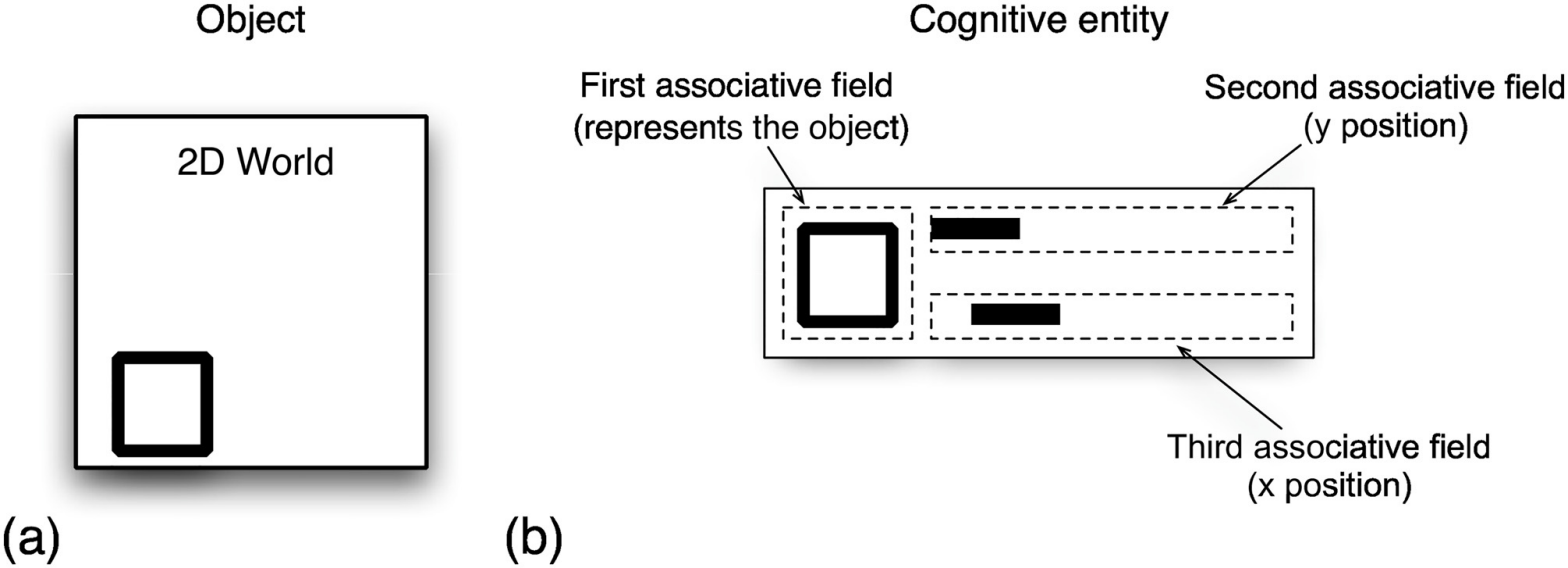
Representation of an object in a 2D world. (a) 2D world. (b). The identity of an object is represented in the first associative field by a binary pattern which is normalized for size and orientation. Its location corresponding to the abscissa is represented by a binary vector in the second associative field. The location corresponding to the ordinate is likewise represented by a binary vector in the third associative field of the size of the ordinate of the pictogram representing the state. A binary bar of the size and position of the object in the pictogram of the state represents the location.

According to [11, 12, 14, 27] the identity of an object can be represented by a binary pattern which is normalized for size and orientation. Its location in the *x*-axis is represented by a binary vector of the size of the abscissa of the pictogram representing the object. The location in the *y*-axis is likewise represented by a binary vector of the size of the coordinate of the pictogram representing the object. A binary bar of the size and position of the object in the pictogram of the state represents the location and size (see Fig 4) in each of those vectors. The three vectors that compose the cognitive entity are called associative fields. Each associative field is represented by a binary vector of a fixed dimension; each cognitive entity is formed by the concatenation of the associative fields [11, 12, 14]. A cognitive entity is represented by a binary vector formed by the concatenation of binary vectors which represent the three associative fields [11, 12, 14, 27].

### Associations

**Fig 5:**
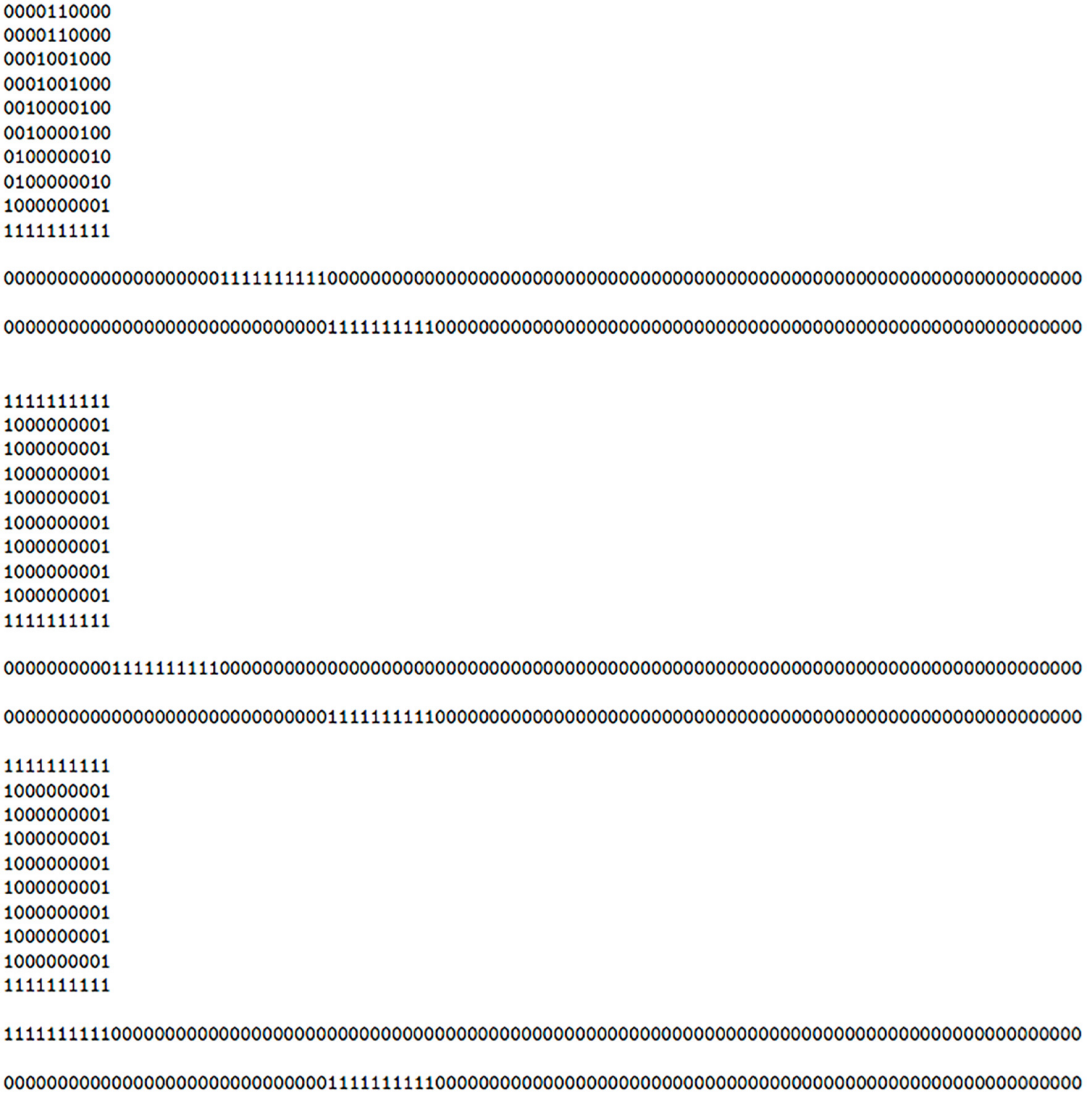
Representation by cognitive entities. The representation of the category “tower” by three cognitive entities.

A cognitive entity is represented by a binary vector formed by the concatenation of binary vectors which represent the three associative fields. A visual category “tower” is represented in the blockworld as shown in Fig 3. It corresponds to a set of prototypical visual objects at certain position. The address and retrieved vectors are represented by a binary vector formed by the concatenation of three binary sub-vectors which represent the cognitive entities. Both the question and the answer vectors have dimension 900 because each cognitive entity is described by a binary vector of dimension 300 (= *p*). The representation of the category “tower” is shown in Fig 5. Associations representing different positions of the category “tower” are learned by the associative memory and can be recognized later despite the presence of noise. Ten associations representing ten different positions of the category “tower” are learned by the associative memory. After learning is complete, a weight matrix of dimension *n* = 900 emerges; the weight matrix consists of three parts, each of size *p* = 300 and *n* = 3 ⋅ *p* = 3 ⋅ 300.

### Retrieval

**Fig 6:**
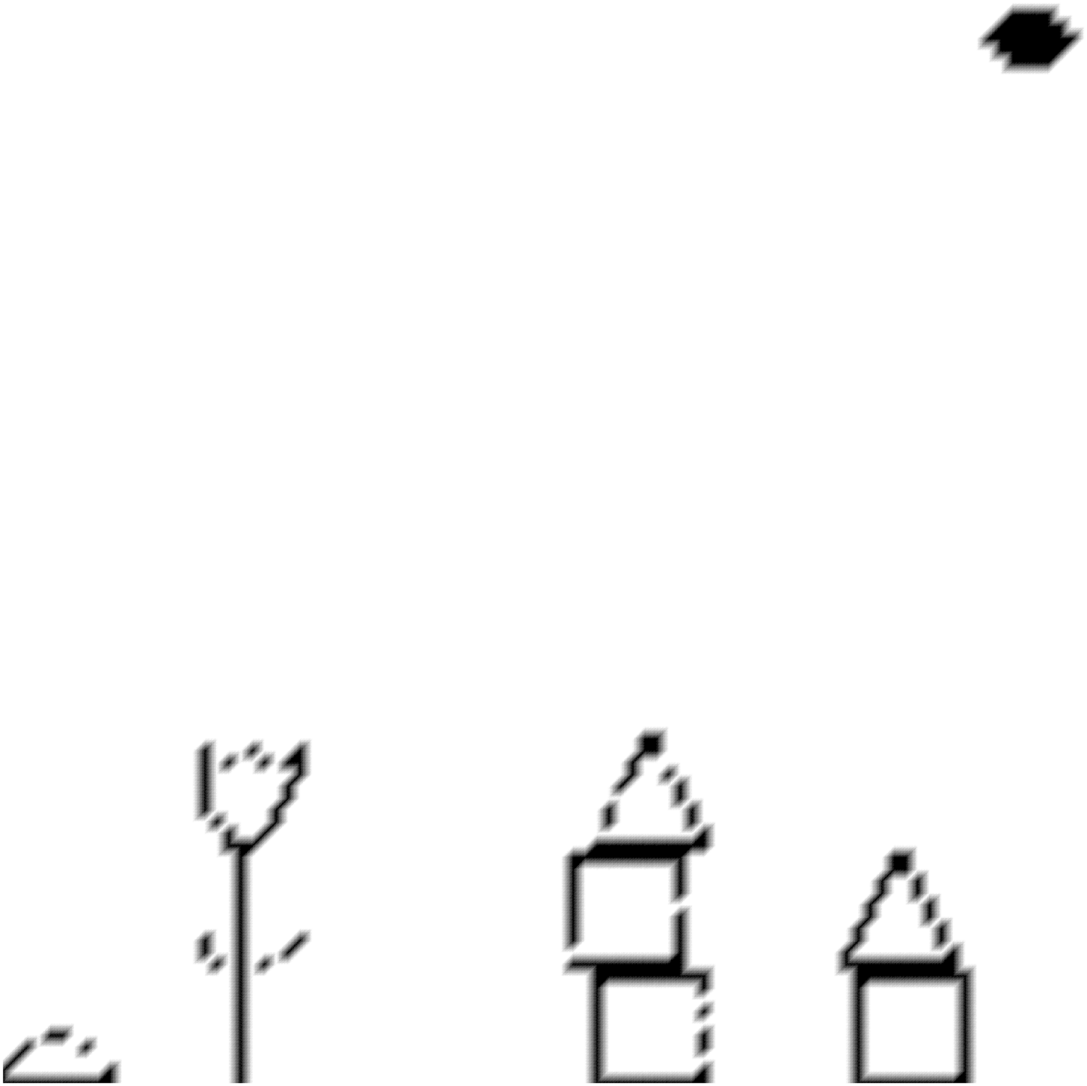
Visual representation by a pictogram. Visual representation of the world with nine different objects and with the category “tower” including noise. The state of the world is described by a pictogram of 100 × 100 pixels; the pictogram is represented by *N* = 10 cognitive entities, and the category tower is described by *M* = 3 cognitive entities.

In the following example, we indicate the recognition of the category tower (Fig 3) from the pictogram in Fig 6 that is represented by nine different objects. To determine the visual category present, a familiar pattern is determined. During familiarity discrimination, there is no need to extract the entire answer pattern. The corresponding vector values of

$$\begin{array}{c} 720=\frac{10!}{(10-3)!} \end{array}$$

combinations are determined, and the category tower is identified from the noisy input by using Eqs 11 and 13 with threshold *t* = 0.87. After determining the correct input vector from the 720 possible combinations, the associative memory is queried, and the answer vector representing the category tower without noise is determined (see Fig 3).

## Results and Discussion

### Quantum Hybrid Algorithm for Sub-symbolic Binding

In our proposed hybrid approach, we will use Eqs 11 and 13 to build an oracle and Grover’s algorithm to speed up the combination of Eq 1. By doing so, we can overcome the RD problem. After determining the correct input vector from all possible combinations, the classical associative memory is queried, and the answer vector representing the category is obtained.

To simplify the computational process, we can approximate *L* by permutations with repetition

$$\begin{array}{c} P=N^{M} \end{array}$$ (15)

where *P* > *L*. Based on this simplification, we develop a reversible circuit that generates all possible *M* permutations with repetitions of the *N* objects. The register |*x*〉 should represent the superposition of the *N* different objects that represent a visual scene; the read time would be *O*(*N*). We start with the assumption *M* = 1. In this case, *P* = *N*, and the superposition corresponds to *N* objects. Each represented object is identified by a unique address with a value ranging from 1 to *N*. The task involves loading the corresponding representation of the object having a given address *α* into the register |*x*〉 by a reversible circuit. This task is equivalent to the process of initializing the amplitude distribution of a quantum state, as described in [28, 29]. However, instead of describing the distribution by using a quantum circuit, we load the required values from a given set by using a reversible circuit.

#### Reversible load

Each object is represented by a vector (of size *p*) describing the object, the corresponding address and a flag that is set to 0,

$$\begin{array}{c} scene=\left(z_{1},address_{1},flag_{1}\right),\left(z_{2},address_{2},flag_{2}\right),\cdots ,\left(z_{N},address_{N},flag_{N}\right) \end{array}$$

$$\begin{array}{c} scene=\left(z_{1},1,0\right),\left(z_{2},2,0\right),\cdots ,\left(z_{N},N,0\right). \end{array}$$

For a given address *α*, the corresponding address is determined, and the flag is set to one. This task can be performed by a reversible circuit by subtracting the given address *α* from the unique address, verifying whether the difference is zero and checking whether the result is zero. After this operation, the flag is equal to one. The loading operation is performed by the reversible circuit *load*(*α*, **scene**)

$$\begin{array}{c} x_{address}=load\left(\alpha ,scene\right)=:\left(z_{1}\wedge flag_{1}\right)\vee \left(z_{2}\wedge flag_{2}\right)\vee \cdots \vee \left(z_{N}\wedge flag_{N}\right). \end{array}$$

using *N* ⋅ (*N*−1) ⋅ *p* reversible gates. The reversible circuit corresponds to the reversible operator *U*_*load*_

$$\begin{array}{c} |x_{address}\rangle |\alpha \rangle |scene\rangle =U_{load}\left|0\cdots 0\rangle |\alpha \rangle |scene\right\rangle \end{array}$$ (16)

that loads the object representation from *scene* to a given address *α* and the resulting waste bits into |**x**_*address*_〉. Each binary ∧ and ∨ generates one waste bit due to the reversible implementation by a Toffoli gate.

#### Superposition

The superposition of the address is generated by a Hadamard gate with

$$\begin{array}{c} \nu =2^{\left\lceil log_{2}N\right\rceil }\geq N \end{array}$$ (17)

and

$$\begin{array}{c} |\alpha^{*}\rangle :=H_{\nu }\underset{\nu \;\text{bits}}{\underset{︸}{\left|00\cdots 0\right\rangle }}=\underset{\text{amplitude}}{\underset{︸}{\frac{1}{\sqrt{2^{\nu }}}}}\sum_{x=0}^{2^{\nu }-1}\underset{\nu \;\text{bits}}{\underset{︸}{\left|x\right\rangle }}. \end{array}$$ (18)

In the case *ν* > *N* the scene is represented by *ν*−*N* additional waste objects.

#### Parallel load

We can perform a parallel loading of the corresponding representation of the object with a certain address represented in the superposition of addresses |α*〉 along with the resulting waste bits into the register |*ψ*_x_〉 by using the reversible operator *U*_*load*_.

$$\begin{array}{c} U_{load}\left|0\cdots 0\rangle \right|\alpha^{*}\rangle |scene\rangle =U_{load}\left|0\cdots 0\right\rangle ⊗H_{\nu }\left|0\cdots 0\rangle |scene\right\rangle \end{array}$$ (19)

$$\begin{array}{c} |\psi_{x}\rangle |\alpha^{*}\rangle |scene\rangle =U_{load}\left|0\cdots 0\rangle \right|\alpha^{*}\rangle |scene\rangle \end{array}$$ (20)

The register |*ψ*_x_〉 is in superposition because |α*〉 is in superposition. Further, it is entangled with the register |α*〉 that acts as a pointer to the values represented in |**scene**〉. Because |**scene**〉 is equal in each superposition, it is not entangled with |*ψ*_x_〉 and |α*〉.

#### Tensor operation

For *M* > 1 we simply tensor |*ψ*_x_〉|α*〉 *M* times

$$\begin{array}{c} \underset{M\;\text{times}}{\underset{︸}{|\psi_{x}\rangle |\alpha^{*}\rangle ⊗|\psi_{x}\rangle |\alpha^{*}\rangle ⊗\cdots ⊗|\psi_{x}\rangle |\alpha^{*}\rangle }}\left|scene\right\rangle \end{array}$$ (21)

For simplicity we define

$$\begin{array}{c} |x^{*}\rangle :=\underset{M\;\text{times}}{\underset{︸}{|\psi_{x}\rangle |\alpha^{*}\rangle ⊗|\psi_{x}\rangle |\alpha^{*}\rangle ⊗\cdots ⊗|\psi_{x}\rangle |\alpha^{*}\rangle }} \end{array}$$ (22)

it follows

$$\begin{array}{c} |x^{*}\rangle |scene\rangle =\underset{M\;\text{times}}{\underset{︸}{|\psi_{x}\rangle |\alpha^{*}\rangle ⊗|\psi_{x}\rangle |\alpha^{*}\rangle ⊗\cdots ⊗|\psi_{x}\rangle |\alpha^{*}\rangle }}\left|scene\right\rangle . \end{array}$$ (23)

The register |x*〉 represent *P*′ superpositions of all possible objects *M*-permutations with repetition of the *N* objects as well as the entangled addresses and waste bits with

$$\begin{array}{c} P^{\prime }=\nu^{M}={2^{\left\lceil log_{2}N\right\rceil }}^{M}\geq N^{M}. \end{array}$$ (24)

The complexity of the operation is of size *O*(*N* ⋅ *M*).

### Quantum Oracle for Familiarity Discrimination

The quadratic form can be simplified because the input vector **x** and the weight matrix *W* are binary

$$\begin{array}{c} net=\sum_{i=1}^{n}\sum_{j=i}^{n}w_{ij}\wedge x_{i}\wedge x_{j}. \end{array}$$ (25)

This operation requires 2 ⋅ *n*^2^ AND operations and *n*^2^ full adder gates. A full adder is usually a component in a cascade of adders that adds together several bits, for example a four byte adder. A full adder adds two bits together with a bit carried in form another full adder. Its output is one bit together with the bit that is carried out to another full adder (overflow bit).

#### Reversibles circuit for familiarity discrimination

A reversible full adder can be build out of Toffoli gates. It itself can be represented as the Peres full adder gate [30]. The Peres full adder gate does not change the first input bit *x*_1_. The operation is described by the following mapping on three input bits *x*_1_, *x*_2_, *x*_3_ with **B** = {0, 1}

$$\begin{array}{c} P:B^{4}\to B^{4}:P\left(x_{1},x_{2},x_{3},x_{4}\right)=\left(y_{1},y_{2},y_{3},y_{4}\right) \end{array}$$

$$\begin{array}{c} P\left(x_{1},x_{2},x_{3},x_{4}\right)=\left(x_{1},\left(x_{1}⊕x_{2}\right),\left(x_{1}⊕x_{2}⊕x_{3}\right),\left(x_{1}⊕x_{2}\right)\wedge x_{3}⊕x_{1}\wedge x_{2}⊕x_{4}\right) \end{array}$$

It computes the full adder operation with the ancilla (fixed) bit *x*_4_ set to 0

$$\begin{array}{c} P\left(x_{1},x_{2},x_{3},0\right)=\left(x_{1},\left(x_{1}⊕x_{2}\right),\underset{\text{sum}}{\underset{︸}{\left(x_{1}⊕x_{2}⊕x_{3}\right)}},\underset{\text{carry}}{\underset{︸}{\left(\left(x_{1}⊕x_{2}\right)\wedge x_{3}⊕x_{1}\wedge x_{2}\right)}}\right) \end{array}$$

bit number *x*_1_ and *x*_2_ are the operands and *x*_3_ is the bit carried in from the previous less significant stage.

To determine if the question vector **x** was stored in the associative memory we subtract from the threshold *net* from *t*

$$\begin{array}{c} sim=\left\{\begin{array}{cc} 1 & \;(t-net)<\;\text{0}\;\\ 0 & \;\text{otherwise} \end{array}\right. \end{array}$$ (26)

with the threshold

$$\begin{array}{c} t:=T^{2}. \end{array}$$ (27)

A full subtractor can be designed using the same approach as that for an adder. Three bits are involved in performing the subtraction for each bit of the difference: the minuend *x*_1_, the subtrahend *x*_2_, and the borrow in from the previous (less significant) bit order position *x*_3_. The full subtractor gate does not change the first input bit *x*_1_. It performs the full subtractor operation with the ancilla (fixed) bit *x*_4_ set to 0.

$$\begin{array}{c} S\left(x_{1},x_{2},x_{3},0\right)=\left(x_{1},\left(x_{1}⊕x_{2}\right),\underset{\text{subtraction}}{\underset{︸}{\left(x_{1}⊕x_{2}⊕x_{3}\right)}},\underset{\text{borrowed}\;\text{bit}}{\underset{︸}{\left(\neg x_{1}\wedge \left(x_{2}\vee x_{3}\right)\vee x_{2}\wedge x_{3}\right)}}\right) \end{array}$$

where bit numbers *x*_1_ and *x*_2_ are the operands, and *x*_3_ is the borrowed bit. The value of *sim* can be determined from the borrow bit in the result; if the borrow bit is equal to one, then *sim* = 1, otherwise *sim* = 0. This operation requires *n*^2^ full subtractor gates. The reversible familiarity discrimination is feasible because it requires 4 ⋅ *n*^2^ reversible gates (2 ⋅ *n*^2^ and gates, *n*^2^ full subtractor gates and *n*^2^ full adder gates).

The reversible circuit corresponds to the reversible operator *U*_*Sim*_. Permutation is a reversible operation; therefore, |x*〉 can be rearranged in such a way that the operator *U*_*Sim*_ accesses only the object representation. In addition to the object description, *U*_*Sim*_ requires the weight matrix representation as a binary vector **w**. The results or the sums, ∧ operations, subtraction operations and waste bits are mapped into the register |**result**⟩.

$$\begin{array}{c} \left|result\rangle \right|x^{*}\rangle |scene\rangle |w\rangle =U_{Sim}\cdot \left|0\cdots 0\rangle \right|x^{*}\rangle |scene\rangle |w\rangle \end{array}$$ (28)

A single bit, the borrow bit, determines the familiarity discrimination and whether the subtraction is negative; if the borrow bit is equal to one, then *sim* = 1, otherwise *sim* = 0. We use a circuit *U*_*CNOT*_ composed of a controlled not gates to copy the result represented in the borrow bit into a single bit *sim*.

$$\begin{array}{c} \left|sim\rangle |result\rangle \right|x^{*}\rangle |scene\rangle |w\rangle =U_{CNOT}\cdot \left|0\rangle |result\rangle \right|x^{*}\rangle |scene\rangle |w\rangle \end{array}$$ (29)

In quantum computation it is not possible to reset value of the register |**result**⟩. Instead we un-compute the former operations of *U*_*Sim*_ by applying the $U_{Sim}^{-1}$ operator.

$$\begin{array}{c} \left|sim\rangle |0\cdots 0\rangle \right|x^{*}\rangle |scene\rangle |w\rangle =U_{Sim}^{-1}\cdot \left|sim\rangle |result\rangle \right|x^{*}\rangle |scene\rangle |w\rangle \end{array}$$ (30)

After these operations, the result of the familiarity operation is represented in the bit |*sim*〉, which is in the basis state |1〉 or |0〉. The registers |*sim*〉 and |x*〉 are entangled. The remaining registers |**scene**〉, |**W**〉 and |0⋯0〉 are not entangled because they are equal. We simplify the notation by defining the global operator *U_F_*. The unitary operator *U*_*F*_ represents our quantum oracle that marks the solution by the bit *sim*; it is composed of the operators *U*_*Sim*_, *U*_*CNOT*_ and $U_{Sim}^{-1}$. Its operation corresponds to

$$\begin{array}{c} \left|sim\rangle |0\cdots 0\rangle \right|x^{*}\rangle |scene\rangle |w\rangle =U_{F}\cdot \left|0\rangle |0\cdots 0\rangle \right|x^{*}\rangle |scene\rangle |w\rangle . \end{array}$$ (31)

### Grover’s iteration

The number of solutions (in our case, categories) can be determined efficiently by Quantum Fourier Transform [31], [32].

#### One category present

If one solution is present, Grover’s amplification [33], [34], [35], [36], [33], [33] algorithm requires

$$\begin{array}{c} O\left({\left(2^{\left\lceil log_{2}N\right\rceil }\right)}^{\frac{M}{2}}\right)\approx O\left(N^{\frac{M}{2}}\right)=O\left(\sqrt{N^{M}}\right) \end{array}$$ (32)

applications of the quantum oracle [32], [31] *U_F_*

$$\begin{array}{c} \left|sim\rangle |0\cdots 0\rangle \right|x^{*}\rangle |scene\rangle |w\rangle =U_{F}\cdot \left|0\rangle |0\cdots 0\rangle \right|x^{*}\rangle |scene\rangle |w\rangle . \end{array}$$ (33)

to determine the correct input vector representing a category that was stored in the associative memory with high probability. After determining the correct input vector, the classic Wilshaw’s associative memory is queried, and the vector representing the category without noise is determined.

#### Several categories present

For *r* possible categories, only one category is determined with the cost.

$$\begin{array}{c} O\left({\left(\frac{2^{\left\lceil log_{2}N\right\rceil }}{r}\right)}^{\frac{M}{2}}\right) \end{array}$$ (34)

To determine all of the categories, the algorithm can be repeated several times until *r* different input vectors representing the categories are measured. An alternative approach would be to unlearn the associative memory specified by *W* from the already recognized category [37].

### Cost Analysis

The reversible load requires *N* ⋅ (*N*−1) ⋅ *p* reversible gates and the reversible familiarity discrimination requires 4 ⋅ *n*^2^ reversible gates with

$$\begin{array}{c} 4\cdot n^{2}=4\cdot {\left(p\cdot M\right)}^{2}. \end{array}$$

Both operation are feasible and require the same number of gates as a classical application. The time complexity of the quantum hybrid algorithm for sub-symbolic binding is significant better then the classical (naïve) approach. For simplification of the computational process *L* is approximated by *P* permutations with repetition for one present category. The costs of the algorithm are

$$\begin{array}{c} O\left(\sqrt{N^{M}}\right)=O\left(\sqrt{N^{M}}\right)+O\left(N\cdot M\right) \end{array}$$ (35)

with

$$\begin{array}{c} {\left(2^{\left\lceil log_{2}N\right\rceil }\right)}^{\frac{M}{2}}<<L=Perm\left(N,M\right)<N^{M}=P. \end{array}$$ (36)

## Conclusion

Using Grover’s algorithm, we could achieve a quadratic speed-up without requiring the output states to be quantum states. For example, let us consider a range query vector **y** from a collection of *N* vectors,

$$\begin{array}{c} x_{1},x_{2},x_{3},\cdots ,x_{N} \end{array}$$

*all* vectors **x**_*i*_ that are *ϵ*-similar according to the distance function *d* are searched

$$\begin{array}{c} d(x_{i},y)<\epsilon . \end{array}$$ (37)

Assuming that the collection of vectors is represented in a superposition, e.g., in |*ψ*〉, the cost to determine one possible *ϵ*-similar vector would be $O(\sqrt{N})$ using the quantum oracle described by Eq 37. The assumption that the speed-up is quadratic is not realistic in practice due to the RD problem; we are required to read *N* data points and can query only once because of the collapse during measurement (destruction). If the collection of vectors is represented in a superposition, e.g., in |*ψ*〉, oould we copy |*ψ*〉 before the measurement? An operation that would produce a copy of an arbitrary quantum state such as |*ψ*〉 is not possible; we cannot copy non-basis states because of the linearity of quantum mechanics. Due to the collapse during measurement (destruction), no advantage over a classical algorithm can be achieved.

Instead of being represented by units in superposition [6–8], the quantum associative memory is described by a quantum oracle for familiarity discrimination. The input represents permutations of visual objects by superposition. This architecture reduces the computational complexity and represents the relation between the associative memory and a quantum algorithm by a familiarity discrimination task. The proposed approach is a hybrid algorithm because after determining the correct input vector, the classic Wilshaw’s associative memory is queried, and the vector representing the category without noise is determined.

Some classical improvements to the classical naive approach were suggested in [11, 12] and involved a more complicated architecture that does not scale up. It is an open question whether such a quantum hybrid approach has any relation with the human brain. It should be noted that the associative memory performs a classical operation and that the quantum aspect involves a familiarity oracle that performs a subconscious determination of the correct input.

In this paper, we present a method to map an algorithm with an associative memory to Grover’s amplification algorithm. This mapping may serve as an inspiration for other related algorithms and problems.
